# PP2Cδ Controls the Differentiation and Function of Dendritic Cells Through Regulating the NSD2/mTORC2/ACLY Pathway

**DOI:** 10.3389/fimmu.2021.751409

**Published:** 2022-01-07

**Authors:** Nianyin Lv, Sufeng Jin, Zihao Liang, Xiaohui Wu, Yanhua Kang, Lan Su, Yeping Dong, Bingwei Wang, Tonghui Ma, Liyun Shi

**Affiliations:** ^1^ Department of Immunology, Nanjing University of Chinese Medicine, Nanjing, China; ^2^ Department of Clinical Laboratory, The Children’s Hospital, Zhejiang University School of Medicine, National Clinical Research Center for Child Health, Hangzhou, China; ^3^ Key Lab of Inflammation and Immunoregulation, Hangzhou Normal University School of Medicine, Hangzhou, China; ^4^ Institute of Translational Medicine, Zhejiang Shuren University, Hangzhou, China; ^5^ College of Medicine and Integrated Medicine, Nanjing University of Chinese Medicine, Nanjing, China

**Keywords:** autoimmunity, dendritic cells, differentiation, PP2Cδ, mTORC2

## Abstract

Dendritic cells (DCs) are recognized as a key orchestrator of immune response and homeostasis, deregulation of which may lead to autoimmunity such as experimental autoimmune encephalomyelitis (EAE). Herein we show that the phosphatase PP2Cδ played a pivotal role in regulating DC activation and function, as PP2Cδ ablation caused aberrant maturation, activation, and Th1/Th17-priming of DCs, and hence induced onset of exacerbated EAE. Mechanistically, PP2Cδ restrained the expression of the essential subunit of mTORC2, Rictor, primarily through de-phosphorylating and proteasomal degradation of the methyltransferase NSD2 *via* CRL4^DCAF2^ E3 ligase. Loss of PP2Cδ in DCs accordingly sustained activation of the Rictor/mTORC2 pathway and boosted glycolytic and mitochondrial metabolism. Consequently, ATP-citrate lyse (ACLY) was increasingly activated and catalyzed acetyl-CoA for expression of the genes compatible with hyperactivated DCs under PP2Cδ deletion. Collectively, our findings demonstrate that PP2Cδ has an essential role in controlling DCs activation and function, which is critical for prevention of autoimmunity.

## Introduction

Dendritic cells (DCs) have been recognized as the most potent antigen-presenting cells with the potential to initiate and orchestrate immune responses (1). Several subsets of DCs have been identified to assume distinct functions in a given setting. Essentially, plasmacytoid DCs (pDCs) are specialized in controlling viral infection *via* producing type I interferon (IFN)-I, whereas conventional DCs (cDCs) play a pivotal role in presenting antigens and stimulating naive T cells. Upon sensing pathogenic signals through pattern-recognition receptors, cDCs undergo the differentiation and activation program, adopting the maturated phenotype with expression of signature surface markers, production of cytokines and chemokines, and migration to lymph nodes to initiate antigen-specific T cell responses (2). The maturation and activation status of DCs has a key role in determining immunological activation or tolerance, deregulation of which may result in immunological disorders such as autoimmune diseases (3, 4).

The development and activation of DCs is a coordinated process with the integration of extracellular signals and intracellular pathways. It is also a metabolically adaptive process requiring bioenergetics and biosynthesis for cell expansion and effector function. The mammalian target of rapamycin (mTOR), a conserved serine/threonine kinase, is a key regulator in both signaling transduction and metabolic sensing, and hence vital for cell fate decisions. Through phosphorylating the downstream ribosomal protein S6, mTOR mediates a wide range of essential biological processes such as ribosome biogenesis, protein translation, and cell mass control modulates (5, 6). mTOR encompasses mTOR complex 1 (mTORC1) and mTORC2, two multi-protein complexes distinguished by the scaffolding proteins, namely, the regulatory associated protein of mTOR (RAPTOR) and rapamycin-insensitive companion of mTOR (RICTOR) (7, 8). Emerging evidences have shown that immune cells, including DCs, act through mTOR pathway to sense antigenic or metabolic signals and couple them with the environmental cues to instruct cell differentiation. Disruption of mTOR signaling proves to impede the maturation and activation of DCs and affect T cell stimulation (9, 10). By contrast, sustained activation of mTORC1 by deletion of the upstream inhibitor tuberous sclerosis 1 (TSC1) caused spontaneous DCs maturation and deregulated T cells induction (11, 12). Compared with the well-appreciated role of mTORC1 in immune regulation, our current understanding of mTORC2 is relatively rudimentary.

mTORC2 is an obligatory activator of AGC subfamily kinases capable of regulating cell proliferation, metabolism, and cytoskeleton reorganization. The kinase can phosphorylate Akt at S473 and promote the activation of the downstream effectors such as NDRG1, FoxO1, and GSK3β (13). Studies have shown that mTORC2/Akt pathway is indispensable for the induction of immune and inflammatory response, and interruption of mTORC2 pathway is proposed to account for the action modes of some critical factors (14–17). Notably, recent studies indicate that mTORC2 activates ATP-citrate lyase (ACLY) and promotes acetyl-CoA generation, which subsequently fuels histone acetylation to facilitate the expression of genes essential for the differentiation of somatic cells and immune cells such as macrophages (18–20). Given its importance in metabolic-epigenetic reprogramming of cellular identities, mTOR signaling is tightly controlled by multiple layers of regulators. Among them, the kinase-phosphatase family members like tuberous sclerosis complex subunit 1/2 (TSC1/2), AMP-activated protein kinase (AMPK), liver kinase B1 (LKB1), and phosphatase and tension homolog (PTEN) have been identified to play a vital role in controlling mTOR signaling (12, 21–24).These kinase/phosphatases, in addition to (de)phosphorylating the substrates directly, may also co-operate with other factors such as E3 ubiquitin ligases or epigenetic modifiers to control the levels of mTOR signaling components (25).

Protein phosphatase 2C delta (PP2Cδ) is a serine/threonine phosphatase belonging to the PP2C family. It targets a network of stress-related substrates such as p53, p38, Chk1/2, ATM, and Akt and mediates the regulation of DNA damage response (26, 27). PP2Cδ is also essential for a variety of vital cellular processes, including tumorigenesis, aging, metabolism, and immunity (28). Accumulating evidences have demonstrated that PP2Cδ exerts the immune regulatory function primarily through its phosphatase activity (29, 30). For instance, PP2Cδ is crucial for differentiation of antigen-independent B cells and maturation of thymus T cells (31, 32). Also, it plays an indispensable role in regulating the development of granulocytes through regulating the p38MAPK-STAT1 pathway (33). PP2Cδ serves as a critical regulator for Th9 cells development and promotes allergy pathogenesis *via* modulating the JNK-c-Jun/c-Fos pathway (34). Despite the well-appreciated role of PP2Cδ in governing immune cell differentiation, its function in DCs activity is yet to be explored. On the other hand, although PP2Cδ has been demonstrated to act through the mTORC1 pathway to impinge hepatocytes and hematopoietic stem cells (HSCs) development (35, 36), its relevance to mTORC2, the other key branch of mTOR signaling, still remains elusive.

In the present study, we identify the phosphatase PP2Cδ as a novel key factor in regulating DCs activation and function through modulating the Rictor/mTORC2 pathway. Ablation of PP2Cδ profoundly enhanced DCs maturation and caused their excessive activation, leading to abnormal Th1/Th17 differentiation and exacerbated EAE pathology. At the molecular level, PP2Cδ restrained the expression of Rictor, the critical component of mTORC2, through de-phosphorylating and hence facilitating proteasomal degradation of the methyltransferase NSD2 *via* CRL4^DCAF2^ E3 ligase. Conversely, de-repression of NSD2 upon PP2Cδ loss led to the elevated Rictor/mTORC2 pathway, leading to augmented mitochondrial oxidative phosphorylation and aerobic glycolysis in DCs. The enhanced mTOR pathway further induced ACLY to catalyze acetyl-CoA for histone acetylation and thereby facilitated the expression of genes characterizing hyperactivated DCs. Thus, our study unveils a previously unknown role for PP2Cδ in regulating DC activation and function and establishes the importance of the Rictor/mTORC2/ACLY axis in metabolic-epigenetic control of immune homeostasis.

## Materials and Methods

### Antibodies and Reagents

Anti-AKT antibody (4691S), antibody to AKT phosphorylated at Ser473 (4060S), antibody to AKT phosphorylated at Thr308 (13038), anti-mTOR antibody (2983S), antibody to mTOR phosphorylated at Ser2448 (5536S), anti-PP2Cδ antibody (11901S), anti-FoxO1 antibody (2880), anti-Rictor antibody (9476S), anti-Raptor antibody (2280S), anti-H3K36me2 antibody (2901S), anti-H3K27me3 antibody (9733S), antibody to ACLY phosphorylated at Ser455 (4331S), anti-Phospho-p70S6Kinase (97596S), anti-p70S6 Kinase (9202s), anti-Phospho-4E-BP1 (2855), anti-4E-BP1 (9644S), anti-H3K9/14Ac antibody (9677S), anti-H3K27Ac antibody (8173S) were from Cell Signaling technology; anti-NSD2 antibody (ab75359), anti-Phospho-serine (ab9332), anti-ACLY (ab40793) were from Abcam; anti-CUL4B antibody (20882-1-AP) was from Proteintech; anti-NDRG1 antibody (abs136385) and antibody to NDRG1 phosphorylated at Thr346 (abs140323) were from Absin.

### Mice

PP2Cδ^-/-^ mice on the C57BL/6J background were kindly provided by Pro. Zhenyu Ju (Institute of Ageing Research, Hangzhou Normal University). OT-II TCR-transgenic mice on the C57BL/6J background were from Pro. Zhijian Cai (School of Medicine, Zhejiang University). Wild-type control mice with same genetic background were used. All mice were housed under specific pathogen-free conditions. Animal experiments were carried out according to the National Institutes of Health Guide for the Care and Use of Laboratory Animal, and approved by the Animal Care and Use Committee of Nanjing University of Chinese Medicine.

### EAE Induction

Wild type and PP2Cδ^-/-^ female mice (8 weeks old) were immunized subcutaneously (s.c.) with 200 μg MOG_35-55_ emulsified in complete Freund’s adjuvant (CFA). Animals were also intraperitoneally (i.p.) injected with 400 ng pertussis toxin (PTX; List labs) at 0 h and 48 h after immunization. The clinical score of EAE was rated using the following scale: 0, no clinical symptoms; 1, tail paralysis; 2, hindlimb weakness; 3, unilateral hindlimb paralysis; 4, complete hindlimb paralysis; 5, moribund or death (37).

### Passive EAE Induction

BMDCs from WT and PP2Cδ^-/-^ mice were treated with 10 ng/mL LPS for 24 h and pulsed with 50 μg/mL MOG_35-55_ for 12 h. 3 x 10^6^ cells were then administered subcutaneously into the flank region of C57BL/6 mice every 5 days for a total of 4 times. The mice were also intraperitoneally (i.p.) injected with 200 ng PTX at 0 h and 48 h after each cell immunization. The clinical scores were recorded after the last immunization (37).

### Histopathological Analysis

Spinal cords from EAE mice were fixed in 4% paraformaldehyde for 2 days. Then, the specimen was embedded in paraffin for sectioning. The paraffin sections were stained with hematoxylin & eosin (H&E) and Luxol Fast Blue (LFB) using standard procedures to evaluate CNS inflammation and demyelination.

### BMDC Generation

Bone marrow (BM) cells isolated from 6 weeks’ WT or PP2Cδ^-/-^ mice were cultured in complete RPMI-1640 medium supplemented with 20 ng/mL rmGM-CSF (PeproTech), and 10 ng/mL IL-4 (PeproTech). After 3 days, half of the medium was removed, and a fresh medium containing GM-CSF and IL-4 was added. On the sixth day, loose semi-adherent cells were collected, washed and subjected to positive selection with CD11c MicroBeads UltraPure (Miltenyi Biotec).

### Mixed Lymphocyte Reaction Assay

BMDCs were stimulated with 10 ng/mL LPS for 24 h and pulsed with 1 μg/mL OVA_323-339_ for 12 h, and then co-cultured with OT-II CD4^+^T cells (labeled with CFSE) to a scale of 1:5 (DC/T cell) for 72 h. The differentiation was identified by flow cytometry and the supernatants were collected for cytokines examination by ELISA.

### Reactive Oxygen Species Detection

Intracellular ROS level was detected using ROS Assay Kit (Beyotime) according to the manufacturer’s instructions. Briefly, DCs, with or without LPS stimulation, were incubated with 10 μM DCFH-DA at 37°C for 20 min. DCFH-DA was hydrolyzed into DCFH by intracellular esterase and further oxidized by ROS to produce fluorescent DCF. The DCF fluorescence was assessed by flow cytometry.

### Cytokine Measurement

Commercially available ELISA kits (DAKEWE) were used to quantify supernatant levels of cytokines in BMDCs and T cells following the manufacturer’s instructions.

### Cell Transfection, Immunoblotting, and Coimmunoprecipitation Assay

Plasmids or siRNA were transfected into cells using X-tremeGENE HP DNA Transfection Reagent (Roche) or X-tremeGENE siRNA Transfection Reagent (Roche), respectively, according to the manufacturer’s instructions. The siRNA sequences are in **Table S1**.

For immunoblotting analysis, cells were lysed with RIPA buffer containing PMSF. Equal amounts of protein were separated on 10% SDS-PAGE and transferred onto Immobilon membranes (Millipore) followed by blocking in Tris-buffered saline with Tween-20 (TBST) containing 5% skim milk (BD). Membranes were then incubated with the indicated primary antibodies overnight, followed by the incubation of fluorescent secondary antibodies. The immunoblotted proteins were visualized with an ECL detection reagent (Yeasen).

For co-immunoprecipitation analysis, cells were lysed with NP-40 lysis buffer containing PMSF. 300 μg of cell lysis were incubated with Dynabeads (1001D, Invitrogen) that were pre-coupled with 3 μg target primary antibodies for 1h at room temperature. Dynabeads-Ab-Ag complex was then collected, washed 3 times and resuspended in elution buffer, and heated for 5 min at 100°C in SDS buffer for immunoblotting.

### Quantitative Real-Time PCR

Total RNA was extracted using TRIzol reagent (Invitrogen), and cDNA was synthesized with the HI Script II One-Step RT-PCR kit (Vazyme). Quantitative RT-PCR was performed using qPCR SYBR Green Master Mix (Yeasen). The mRNA expression level was calculated by applying the 2^-△△Ct^ method. The primer sequences are in **Table S1**.

### Flow Cytometry

For cell surface markers analysis, single-cells in the suspensions, after FcR blocking, were respectively incubated with FITC-anti-CD40 (11-0402-82, eBioscience), APC-anti-CD80 (17-0801-82, eBioscience), PE-anti-CD86 (12-0862-82, eBioscience), PE-anti-MHC Class II (12-5321-82, eBioscience), PerCP-Cyanine5.5-anti-CD11b (45-0112-82, eBioscience), FITC-anti-CD8a (11-0081-82, eBioscience), APC-anti-CD11c (85-17-0128-41, MultiSciences) for 30 min at 4°C. Cells were then washed and detected by flow cytometry.

For intracellular cytokines analysis, splenocytes were incubated in RPMI1640 containing PMA (50 ng/ml), ionomycin (1 μg/mL) (MultiSciences), and Golgi-stop (BD) at 37°C for 6 h. Cells were then washed and incubated with FITC-anti-CD4 (11-0041-81, eBioscience) and APC-anti-CD3 (17-0031-82, eBioscience) for 30 min. After that, cells were fixed and permeabilized with the Fixation/Permeabilization Kit, followed by staining with PE-anti-IFN-γ or PE-anti-IL-17A for 1 h. For regulatory T cell analysis, cells were incubated with FITC-anti-CD4 and PE-anti-CD25 for 30 min, followed by fixation, permeabilization, and staining with APC-anti-Foxp3 (Mouse Regulatory T cell, Invitrogen).

For proliferation assay, DCs, on day 3 and day 6 of culture, were labeled with BrdU for 3 h before analysis. For apoptosis assay, DCs were collected and performed using FITC Annexin V Apoptosis Detection Kit (BD) as per the manufacturer’s instructions.

MitoTracker Green, Mito Tracker Red, and Mito SOX staining were performed according to the manufacturer’s instructions (Invitrogen).

### Chromatin Immunoprecipitation Assay

The chromatin immunoprecipitation (ChIP) assay was carried out using SimpleChIP plus Sonication Chromatin IP Kit according to the manufacturer’s instructions (CST). In short, BMDCs were stimulated with LPS for 24 h and fixed with 1% formaldehyde solution. Solution chromatin was immunoprecipitated with anti-NSD2 (Abcam), H3K9/14Ac, or anti-IgG antibody (CST) respectively. Precipitated DNA and input DNA were assessed by real-time PCR.

### Metabolism Assays

Oxygen consumption rates (OCR) and extracellular acidification rates (ECAR) of BMDCs were analyzed with an XF-96 Extracellular Flux Analyzer (Seahorse Bioscience) as described (38). Intracellular ATP concentrations were measured with an ATP Bioluminescence Assay Kit (Beyotime). Briefly, cells were lysed, centrifuged at 12, 000 g at 4°C for 5 min and mixed with luciferase reagent. Luminescence was detected using a Multiscan Spectrum (PerkinElmer).

For mitochondrial DNA quantification, total DNA were extracted from BMDCs, with or without LPS treatment for 6 h, using the FlexiGene (QIAGEN) as per the manufacturer’s instructions. Three pairs of primers against mtDNA and one pair of primers against genomic DNA were used for amplification. The primer sequences are in **Table S1**.

### Transmission Electron Microscopy

BMDCs were stimulated with or without LPS for 12 h, fixed with 2.5% glutaraldehyde for 2 h, and post-fixed in 1% osmium acid in 0.1 M phosphate buffer at room temperature for 2 h. cell samples were embedded for 48 h after dehydration and penetration. Ultrathin sections (60-80 nm) were then prepared using an ultramicrotome (Leica UC7), stained with 2% uranyl acetate and lead citrate for 15 min. The images were finally captured by the HT7700 transmission electron microscope (HITACHI Co.).

### MTT Assay

The proliferation capacity of BMDCs was detected using MTT assay (YEASEN). DCs were plated at a density of 4000 cells/well in 96-well plates and cultured in complete RPMI-1640 medium supplemented with rmGM-CSF and IL-4 (PeproTech) for six days. Plates of cells were added with 5 mg/mL MTT solution and dissolved in DMSO 3h later. The absorbance was measured at 490 nm.

### Acetyl CoA Assay

The level of acetyl CoA was detected using PicoProbe Acetyl CoA Assay Kit (fluorometric) (ab87546) following the manufacturer’s instructions. BMDCs were suspended in 500 μl of the assay buffer and lysed using a glass homogenizer on ice for 20 passes. The free CoA in samples was quenched and acetyl CoA was converted to CoA, which then formed NADH to interact with PicoProbe. The fluorescence signal was detected at Ex/Em = 535/589 nm with a Multiscan Spectrum (PerkinElmer).

### Statistical Analysis

All of the data, from one representative experiment of three independent experiments, are presented as mean ± SD of three technical replicates. For the animal experiments, the representative results are from one of two or three independent experiments. The statistical significance of the differences between two groups was analyzed with Student’s *t*-test. Multiple group comparisons were performed by two-way ANOVA followed by Bonferroni’s *post hoc t*-test. All of the calculations were performed using the Prism software program for Windows (GraphPad Software). A p value of 0.05 or less was considered statistically significant.

## Results

### PP2Cδ Plays a Pivotal Role in Regulating DCs Development and Maturation

Given the documented importance of PP2Cδ in the immune system (29–34), we sought to further explore its potential role in DCs development and function. For this, we firstly evaluated the impact of PP2Cδ on the *in vivo* generation of DCs. Flow cytometry analysis of splenocytes revealed that the frequencies of conventional DCs (cDCs, CD11c^hi^MHC^+^) and plasmacytoid DCs (pDCs, CD11c^int^B220^+^) were mildly affected by PP2Cδ loss, but their numbers were substantially decreased (**Figures S1A, B**). Within cDCs, PP2Cδ ablation caused a profound decrease in counts of CD8α^-^ cDC2 (**Figure S1B**). The results thus indicated that PP2Cδ was essential for DC development.

**Figure 1:**
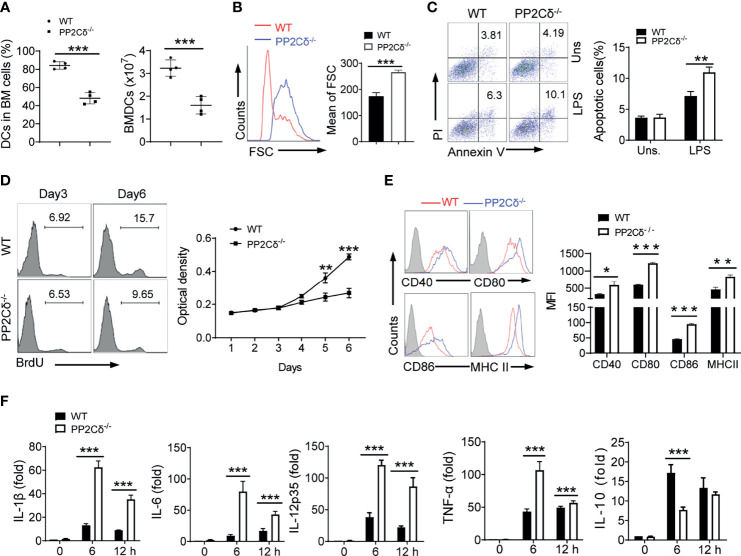
PP2Cδ is essential for DCs development and activation. **(A)** BM cells (input, 4 × 10^7^) from WT or PP2Cδ*^-/-^* mice were cultured in the presence of GM-CSF for 6 d. The percentages and numbers of CD11c^+^MHC^+^ DCs were enumerated. **(B)** Flow cytometry showing size of WT and PP2Cδ^-/-^ DCs at day 6 of culture. FSC, forward scatter. The data represent the mean of FSC. **(C)** Flow cytometry of apoptotic rate of BMDCs with or without LPS (1 μg/mL) stimulation for 8 h; **(D)** Analysis of proliferative potential of DCs by BrdU incorporation or MTT assay; **(E)** Flow cytometry of surface markers of DCs at 12 h post LPS (100 ng/mL) stimulation; **(F)** qPCR assay of the indicated cytokines in DCs; Shown are representative images and the data from three independent experiments are expressed as means ± SD, with two or three technical replicates. *P < 0.05, **P < 0.01, ***P < 0.001 by student’s *t* test.

Next, we detected the role of PP2Cδ in DCs differentiation by exploiting the *in vitro* culture of bone marrow (BM) cells. Strikingly, the frequency and count of CD11c^+^ DCs developed from hematopoietic stem cells (HSCs) were reduced upon PP2Cδ deletion (**Figure 1A** and **Figure S1C**). Few quantities of pDCs were generated in this setting likely due to the inhibitory effect of GM-CSF as described previously (39). We also noted that DCs from PP2Cδ lacking mice, though in the diminished numbers, exhibited much larger cellular mass at the end of the 6d culturing period (**Figure 1B** and **Figure S2A**). The results implied that PP2Cδ^-/-^ DCs might be in highly biosynthetic and metabolic status compare with their counterparts.

Considering that cellular numbers were generally the net outcome of cell demise and replication, we therefore proceeded to examine cellular apoptotic and proliferative rates. Indeed, the apoptotic rate of PP2Cδ^-/-^ DCs, upon LPS stimulation, was markedly elevated compared with that of control cells (**Figure 1C**). This pro-apoptotic effect of PP2Cδ loss was likely due to the increased level of cellular ROS (**Figure S2B**). We further assessed the proliferative rate of DCs by applying bromodeoxyuridine (BrdU) incorporation assay and MTT assay. As shown in **Figure 1D**, PP2Cδ^-/-^ DCs displayed blunted proliferative capability compared with WT cells, particularly at the later development stage. In parallel, PP2Cδ lacking DCs demonstrated to increasingly express pro-apoptotic factors such as Bax and P53, while suppressing that of pro-survival factors like Bcl2 and XIAP, and proliferation-associated factors like CDK1, 2, and 4 (**Figure S2C**). Thus, our data indicated that loss of PP2Cδ led to compromised proliferative and surviving potential of DCs, which likely accounted for the diminished numbers of DCs developed from HSCs.

DCs have been recognized as a key player in regulating immune response or tolerance, which is largely determined by their maturation and activation status (1, 3). We therefore further examined the effect of PP2Cδ on the activation and maturation of DCs. Remarkably, PP2Cδ^-/-^ DCs, compared with WT cells, expressed a much higher level of co-stimulatory molecules including CD40, CD80, CD86, and MHC class II following LPS stimulation (**Figure 1E**). In parallel, the cells produced elevated amounts of proinflammatory cytokines IL-1β, IL-6, IL-12, and TNF-α, and suppressed the expression of the immune regulatory cytokine IL-10 (**Figure 1F**). The data thus revealed an important role for PP2Cδ in controlling DCs maturation and activation, loss of which shifted DCs differentiation into the hyperactivated and proinflammatory phenotype.

### PP2Cδ Loss in DCs Drives Pathogenic Th1/Th17 Differentiation and Hence Exaggerated EAE Onset

**Figure 2:**
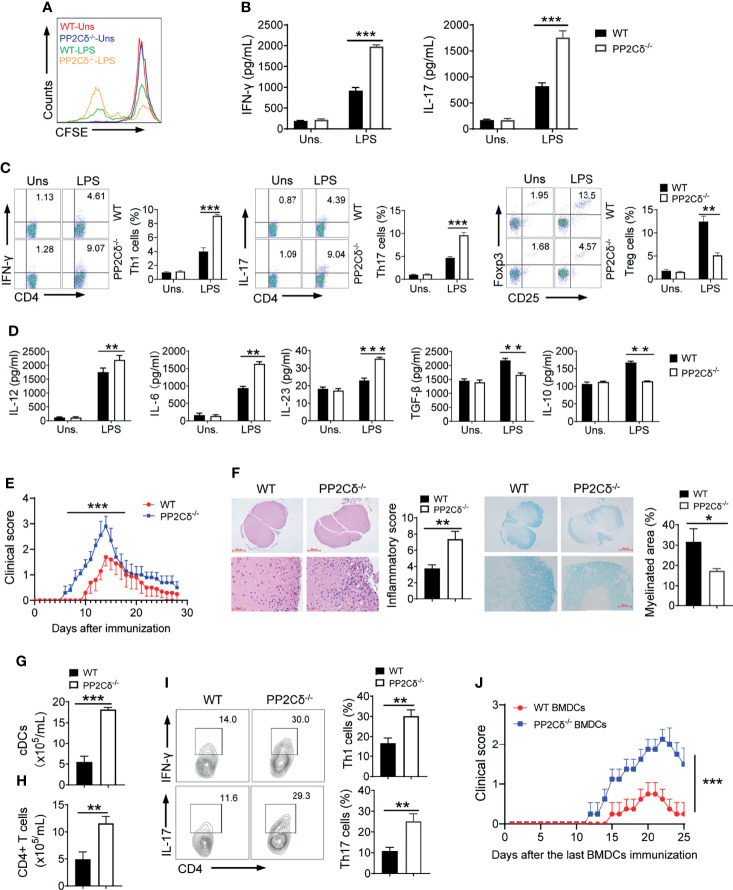
PP2Cδ lacking DCs promote Th1/Th17 differentiation and exaggerated EAE. **(A–D)** DCs were stimulated with LPS and pulsed with OVA peptide, followed by co-culture with OT-II T cells. CFSE dilution of T cells after 72 h co-culture **(A)**; ELISA assay of levels of T cell-secreted cytokines **(B)**; Intracellular staining of signature molecules for Th1, Th17 and Treg cells respectively **(C)**; ELISA assay of the indicated cytokines secreted by DCs **(D)**; **(E–I)** EAE model was established by immunization of mice (n = 10/group) with MOG_35-55_ peptide in CFA, and disease symptoms were daily monitored. Mean clinical EAE scores and cumulative scores **(E)**; H&E and LFB/eosin staining of spinal cord sections **(F)**; Counts of cDCs **(G)** and CD4^+^ T cells **(H)** in spinal cords. Flow cytometry of the frequencies of Th1 and Th17 cells in spinal cords **(I)**; **(J)** LPS-activated and MOG-primed DCs (3 × 10^6^/mice) from WT or PP2Cδ^-/-^ mice were adoptively transferred to C57BL/6 mice (n=4) as described in Methods. Clinical EAE disease scores were documented after last immunization. Shown are representative images and the data are expressed as means ± SD. *P < 0.05, **P < 0.01, ***P < 0.001 by student’s *t* test, shown are data from one representative experiment of two independent experiments.

DCs are the most powerful APCs capable of presenting antigens to naïve CD4^+^ T cells and inducing T cells differentiation and reaction (2). To further define the role of PP2Cδ in DC priming activity, we then exploited the OT-II T cells that are characterized by OVA-specific CD4^+^ T cell receptor (TCR). DCs were firstly matured upon LPS stimulation and pulsed with OVA peptide, followed by co-culture with OT II CD4^+^ T cells. As a consequence, T cells stimulated with PP2Cδ^-/-^ DCs were more proliferative than those induced by WT DCs (**Figure 2A**). These cells secreted much higher levels of IFN-γ and IL-17 (**Figure 2B**). In line, PP2Cδ deficient DCs promoted development of Th1 and Th17 cells while repressing the differentiation of CD4^+^CD25^+^Foxp3^+^ Treg cells (**Figure 2C**). Associated with this, PP2Cδ^-/-^ DCs produced increased levels of IL-12, IL-6, and IL-23, the cytokines essential for instructing differentiation of Th1 and Th17. Conversely, PP2Cδ ablating DCs repressed the expression of tumor growth factor-β (TGF-β) and IL-10 favoring Treg generation (**Figure 2D**). The data thus indicated that loss of PP2Cδ boosted the antigen-presenting activity of DCs and promoted their ability to induce Th1/Th17 development while restricting Treg differentiation.

To further test the *in vivo* functional relevance of these observations, we then exploited experimental autoimmune encephalomyelitis (EAE), a well-appreciated mice model of multiple sclerosis (MS) that are presumably driven by DC-primed Th1 and Th17 cells (4, 40). Remarkably, compared with WT control littermates, PP2Cδ^-/-^ mice displayed accelerated EAE onset and exaggerated disease symptoms upon immunization with myelin oligodendrocyte glycoprotein (MOG)_35-55_ peptide (**Figure 2E**). Exacerbated inflammation and demyelination were observed in lesions of these mice (**Figure 2F**). Consistently, PP2Cδ deficient mice exhibited augmented counts of cDCs and CD4^+^ T cells, along with the elevated ratios of Th1 (IFN-γ^+^) and Th17 (IL-17^+^) cells in spine cords (**Figures 2G–I**). We thus provided the *in vivo* evidences to substantiate the significance of PP2Cδ in controlling DC-driven T cell responses. To exclude the possibility that PP2C might act through other cells than DCs in this setting, we additionally performed passive EAE experiments wherein DCs were pre-activated, loaded with MOG peptide, and transplanted for inducing antigen-specific immune response in mice. Expectedly, mice receiving PP2Cδ^-/-^ DCs displayed earlier onset of EAE with more severe disease symptoms when compared with animals taking WT DCs (**Figure 2J**). Taken together, our data indicated that PP2Cδ played a pivotal role in regulating DCs’ antigen-presenting and T cell stimulatory properties, which was crucial for self-limiting immune response and preventing pathogenic Th1/Th17-driven diseases.

### Loss of PP2Cδ Enhances Mitochondrial Biogenesis and Metabolic Activity of DCs

**Figure 3:**
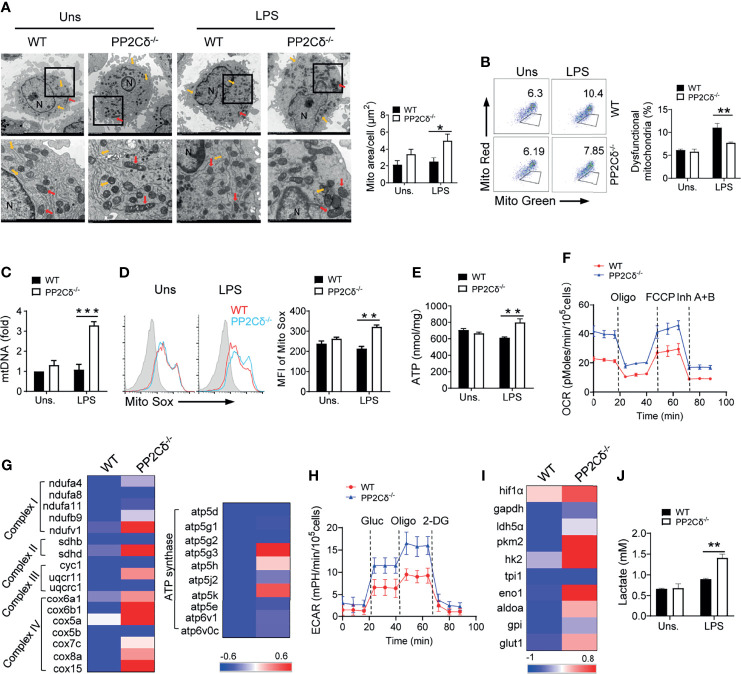
PP2Cδ ablation boosts mitochondrial biogenesis and rewires metabolic program of DCs. BMDCs from WT or PP2Cδ*^-/-^* mice were stimulated with or without LPS (100 ng/mL) and subjected to subsequent analysis. **(A)** Representative transmission electron micrographs (TEM) showing mitochondrial amount, morphology and cristae. The red arrow represents mitochondria and yellow arrow represents ER. The data represent the mitochondria area. **(B)** Flow cytometry of mitochondria staining with MitoTracker Red and MitoTracker green; **(C)** qPCR analysis of mitochondrial DNA level; **(D)** Flow cytometry and quantification of mitochondrial ROS levels by staining with Mito Sox; **(E)** Assay of ATP generation; **(F)** Measurement of oxygen consumption rate (OCR) levels by Seahorse; **(G)** Heatmapping of the genes related with mitochondrial respiration; **(H)** Measurement of extracellular acidification rate (ECAR) by Seahorse; **(I)** Heatmapping of glycolysis-associated genes; **(J)** Assay of lactate production. Shown are representative images and the data from three independent experiments are expressed as means ± SD, with two or three technical replicates. *P < 0.05, **P < 0.01, ***P < 0.001 by student’s *t* test.

Cellular metabolic programs, especially glycolysis and mitochondrial oxidative phosphorylation (OXPHOS), are essential for shaping DC phenotype and functionality (8, 12, 41). Therefore, we further investigated the effect of PP2Cδ on DC metabolism by initially examining the physiology and function of mitochondria, the primary energy supplier as well as the nexus of multiple signaling pathways (42). As shown in **Figure 3A**, while WT control DCs were featured with smaller and round mitochondria dispersed in the cytosol, PP2Cδ lacking DCs displayed larger and elongated mitochondria mostly linked to endoplasmic reticule (ER), indicative of a higher level of metabolic activity (43). In accordance, PP2Cδ^-/-^ DCs exhibited increased mitochondrial mass and less dysfunctional mitochondria, along with the elevated mtDNA level, mtROS release, and ATP generation particularly following LPS stimulation (**Figures 3B–E** and **Figure S3A**). These cells consistently demonstrated an elevated oxygen consumption rate (OCR), the measurement of mitochondrial respiratory capability (**Figure 3F**). Supportively, the expression of genes representative of mitochondrial respiratory chain complex was also augmented in PP2Cδ^-/-^ DCs relative to their counterparts (**Figure 3G**).

Anaerobic glycolysis, though energy inefficient, is critical for synthesizing intermediates and macromolecules to bolster cellular activation and function (41). Compared with WT cells, PP2Cδ^-/-^ DCs displayed significantly enhanced glycolytic activity, as evidenced by elevated extracellular acidification rate (ECAR), increased expression of glycolysis-associated molecules, and augmented lactate production (**Figures 3H–J**). Taken together, our data indicated that PP2Cδ appeared to serve as a metabolic checkpoint to confine mitochondrial respiration and glycolytic metabolism of DCs, loss of which caused exaggerated cellular metabolism compatible with hyperactivated DCs.

### PP2Cδ Controls DCs Activation and Function Through Restraining mTOR2 Activity

**Figure 4:**
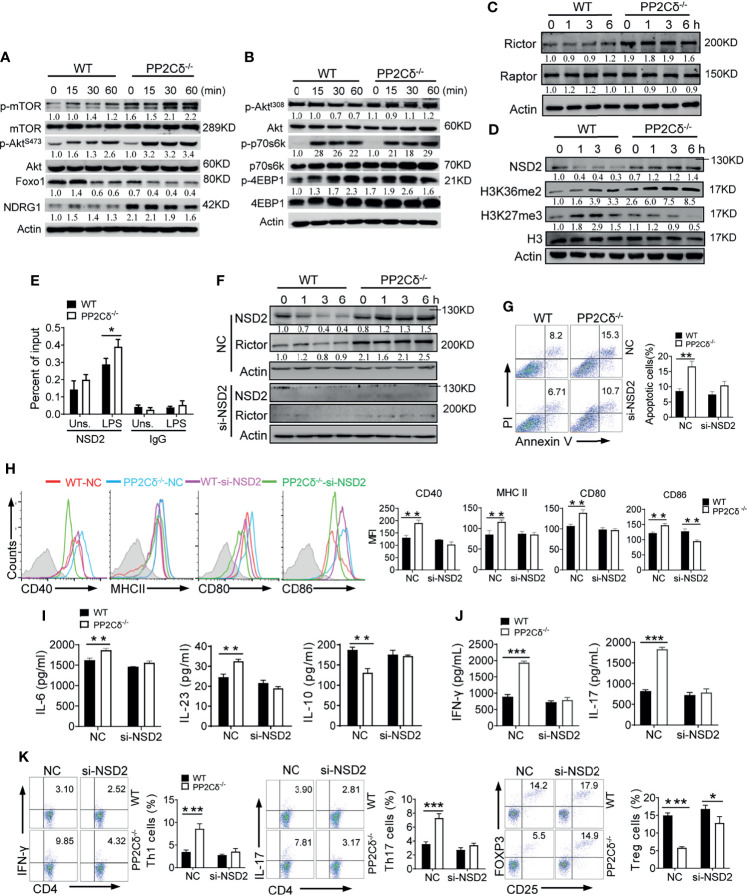
PP2Cδ represses Rictor expression through restraining the level of methyltransferase NSD2. **(A, B)** Immunoblotting analysis for the total or phosphorylated proteins as indicated in WT or PP2Cδ^-/-^ DCs stimulated with LPS (100 ng/mL) for the indicated time periods. **(C, D)** Immunoblotting of Rictor and Raptor **(C)**, or H3K36me2 and H3K27me3 **(D)** in WT or PP2Cδ^-/-^ DCs stimulated with LPS (100 ng/mL) for the indicated time periods. **(E)** ChIP test of NSD2 enrichment at the Rictor locus in WT or PP2Cδ^-/-^ DCs with or without LPS stimulation. IgG as a negative control. **(F)** Immunoblotting of the indicated molecules in WT or PP2Cδ^-/-^ DCs transfected with NSD2-targeted siRNA or non-specific control nucleotides (NC). **(G-K)** WT or PP2Cδ^-/-^ DCs were transfected with NSD2 siRNA or NC, followed by stimulation with LPS. DCs were then subjected to the analysis of apoptotic rate **(G)**; activation markers expression **(H)**, cytokines production **(I)**, T cell-secreted cytokines **(J)** and induction of T cells differentiation **(K)**. The data below the lanes represent the bands densities relative to that of the loading control Actin. Shown are representative images and the data from three independent experiments are expressed as means ± SD, with two or three technical replicates. *P < 0.05, **P < 0.01, ***P < 0.001 by student’s *t* test.

Next, we sought to identify the molecular mechanism underlying PP2Cδ-mediated DC regulation. Given its role in controlling both activity and metabolism of DCs, we wondered whether mechanistic target of rapamycin (mTOR), a master factor integrating cellular metabolism and immunological signaling (6–8), might participate in the action mode of PP2Cδ. Our initial data demonstrated that mTOR was increasingly activated in PP2Cδ^-/-^ DCs relative to WT cells following LPS stimulation. Phosphorylation of Akt at Ser473, indicative of mTORC2 activation, was consistently increased in PP2Cδ^-/-^ DCs. However, phosphorylation of Akt at Thr308 and p70S6K, which was presumably mediated by mTORC1, was not significantly altered. We also observed increased level of NDRG1 and decreased expression of Foxo1, the major signaling events downstream of mTORC2 (21, 44), in PP2Cδ^-/-^ DCs relative to WT cells on LPS stimulation (**Figures 4A, B**). The results thus indicated that loss of PP2Cδ induced the enhanced activation of the mTORC2/Akt pathway in DCs.

We next examined the functional relevance of mTORC2 pathway during DCs activation. Due to the lack of specific mTORC2 inhibitors, we used the mTORC1/2 inhibitor, Torin1, in subsequent studies (**Figure S4A**). Notably, Torin1 treatment largely abolished the effect of PP2Cδ loss on DCs activity, as the key parameters such as the elevated levels of cell size and activation markers in PP2Cδ^-/-^ DCs were substantially reduced to the level comparable to WT cells (**Figures S4B, C**). Also, the augmented production of IL-1β, IL-6, and IL-23 by PP2Cδ^-/-^ DCs was abated while the reduced IL-10 level was resumed upon Torin1 treatment (**Figure S4D**). Significantly, the polarized Th1/Th17 cell differentiation primed by PP2Cδ^-/-^ DCs was reversed following Torin1 treatment, and the generation of Treg cells were restored (**Figures S4E, F**). Collectively, our data indicated that unbridled activation of mTOR, specifically mTORC2 pathway, contributed substantially to deregulated DCs activation and function under PP2Cδ ablation.

### PP2Cδ Controls Rictor Level Through NSD2-Mediated Epigenetic Regulation

As is known, Raptor and Rictor constitute the essential component of mTORC1 and mTORC2 respectively to drive distinct pathways and exert differential effects. In line with the observation that PP2Cδ specifically suppressed mTORC2 pathway in DCs, our data demonstrated that PP2Cδ ablation remarkably elevated Rictor level but marginally affected Raptor expression (**Figure 4C**). The findings prompted us to further investigate how PP2Cδ regulated the level of Rictor. Considering that PP2Cδ was able to co-operate with the epigenetic modulators such as methyltransferase or acetyltransferase to mediate its effector function (21, 45), we then embarked the study on the potential epigenetic regulation by PP2Cδ on Rictor. Of interest, we noted that the expression of NSD2, a histone methyltransferase essential for immunoregulation and tumorigenesis, was remarkably enhanced in PP2Cδ^-/-^ DCs relative to WT cells. Along with this, the enrichment of H3K36me2, an active epigenetic mark catalyzed by NSD2 (46, 47), was increased in PP2Cδ lacking DCs. Conversely, the abundance of H3K27me3, a repressive methylation addition, was shown to be repressed (**Figure 4D**). By performing chromatin immunoprecipitation (ChIP) assay, we further revealed that NSD2 specifically bound to the genomic locus of Rictor in PP2Cδ^-/-^ DCs particularly following LPS stimulation (**Figure 4E**). Accordingly, the expression of Rictor was increased in PP2Cδ^-/-^ DCs, which however was abolished upon NSD2 silencing (**Figure 4F** and **Figure S5A**). The data thus indicated that the methyltransferase NSD2, controlled by PP2Cδ, contributed to the transcriptional induction of Rictor. To be functionally relevant, interference of NSD2 expression largely abrogated the effect of PP2Cδ ablation on DC activity, as demonstrated by a profound reduction in cellular apoptosis, expression of activation markers, secretion of proinflammatory cytokines, as well as by the reversed Th1/Th17 and Treg differentiation in PP2Cδ^-/-^ DCs upon NSD2 knockdown (**Figures 4G-K**).

### PP2Cδ Promotes CUL4^DCAF2^-Mediated NSD2 Degradation *Via* Its Phosphatase Activity

**Figure 5:**
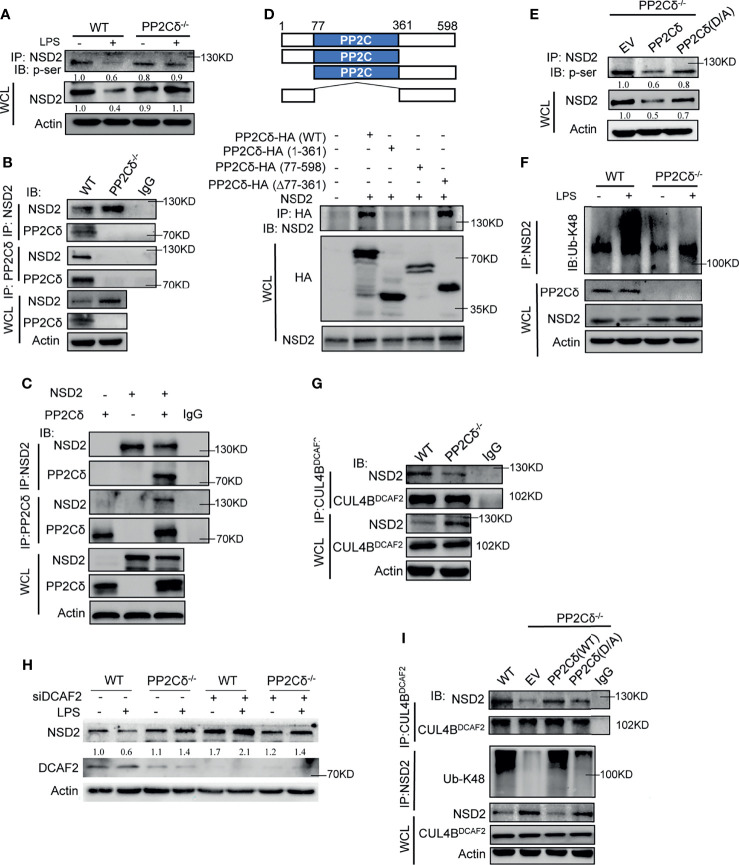
PP2Cδ promotes NSD2 degradation *via* CRL4^DCAF2^ E3 ligase. **(A)** Co-IP analysis of Ser-phosphorylated NSD2 in WT or PP2Cδ^-/-^ DCs with or without LPS stimulation. **(B, C)** Co-IP analysis of the association of NSD2 with PP2Cδ in WT or PP2Cδ^-/-^ DCs **(B)**, or in 293T cells transfected with PP2Cδ- and NSD2-expressing plasmids **(C)**. **(D)** Schematic depiction of full-length and truncated PP2Cδ constructs (upper); Co-IP test of the binding of NSD2 to intact or truncated PP2Cδ fragments as indicated (lower). **(E)** Co-IP examination of Ser-phosphorylated NSD2 in PP2Cδ^-/-^ DCs transfected with empty plasmids, intact or phosphatase-inactivated (D317A) PP2Cδ-expressing plasmids, respectively. **(F)** Co-IP test of K48-linked ubiquitination of NSD2 in WT or PP2Cδ^-/-^ DCs with or without LPS stimulation. **(G)** Co-IP test of the association of CRL4^DCAF2^ with NSD2 in WT or PP2Cδ^-/-^ DCs. **(H)** Immunoblotting of NSD2 in WT or PP2Cδ^-/-^ DCs transfected with DCAF2-targeted siRNA or non-specific control nucleotides (NC). **(I)** Co-IP test of the CRL4^DCAF2^- NSD2 interaction, as well as the level of K48-Ub-linked NSD2 in WT or PP2Cδ^-/-^ DCs that were transfected with the empty, PP2Cδ-expressing or PP2Cδ (D317A)-expressing plasmids as indicated. The data below the lanes represent the bands densities relative to that of the loading control, Actin. Shown are representative images from 2-3 independent experiments.

Then the question to be addressed was how PP2Cδ modulated NSD2 level during DCs activation. As is known, PP2Cδ belongs to the serine/threonine phosphatase family, that has an important role in post-translational regulation of protein stability. We thus set to examine whether PP2Cδ would modulate the stability of NSD2 protein through regulating its phosphorylating status. Strikingly, the level of phosphorylated NSD2 (p-Ser) was shown to be higher in PP2Cδ^-/-^ DCs relative to WT cells following LPS-stimulation (**Figure 5A**). Co-IP assay confirmed that PP2Cδ interacted with NSD2 in WT but not PP2Cδ^-/-^ DCs. This interaction was further corroborated in 293T cells with enforced expression of PP2Cδ and NSD2 (**Figures 5B, C**).

To give a more detailed picture of the PP2Cδ-NSD2 interaction, we then constructed a series of plasmids containing the truncated mutants of PP2Cδ gene. The results showed that, besides the intact fragment, the PP2Cδ truncate lacking B domain (Δ77-361) remained to associate with NSD2. However, the mutants deficient in the N-terminal (77-598) or C-terminal (1-361) failed to bind to NSD2, indicating that the N- and C-terminal but not the catalyze domain (B) of PP2Cδ were required for its association with NSD2 (**Figure 5D**). Nevertheless, the integrity of catalyzing domain proved to be essential for fulfillment of PP2Cδ phosphatase activity, because introduction of the phosphatase-dead mutant (D317A) into PP2Cδ^-/-^ DCs failed to de-phosphorylate NSD2 as the intact PP2Cδ constructs did (**Figure 5E**). Together, the data indicated that PP2Cδ was able to physically associate with NSD2 and mediate the de-phosphorylation effect.

Next, we examined whether PP2Cδ-mediated de-phosphorylation of NSD2 would affect its expressive level. Indeed, NSD2 level was reduced in WT but not PP2Cδ^-/-^ DCs upon LPS stimulation, and this reduction was alleviated by treatment of the proteasome inhibitor MG132 (**Figure S5B**). The result suggested that PP2Cδ might act through the ubiquitin-proteasome pathway to promote NSD2 degradation. Supportively, we observed that K48-linked ubiquitination of NSD2, the prerequisite for proteasomal degradation, was enhanced in WT DCs relative to PP2Cδ^-/-^ cells following LPS stimulation (**Figure 5F**). To further understand this regulatory pathway, our attention was then turned to CUL4^DCAF2^, an E3 ubiquitin ligase that was critically involved in epigenetic regulation and DC modulation (48, 49). Impressively, we confirmed the interaction between CUL4^DCAF2^ and NSD2 using Co-IP assay. This association was shown to be compromised upon PP2Cδ deletion, implying that PP2Cδ-mediated de-phosphorylation of NSD2 was required for this binding (**Figure 5G**). In support of this, the expression of NSD2 was elevated in PP2Cδ^+/+^ DCs upon the deletion of the essential component of CRL4 ligase, DCAF2 (**Figure 5H**). Moreover, the restoration of the intact PP2Cδ but not phosphatase-dead (D317A) expression in PP2Cδ^-/-^ DCs promoted the ligation of CUL4^DCAF2^ to NSD2, leading to the consequent K48-ubiquitination of this protein (**Figure 5I**). The data thus indicated that the phosphatase activity of PP2Cδ was required for the association of CUL4^DCAF2^ with NSD2, and hence the ubiquitination and degradation of NSD2. Of functional relevance, the resumption of wild-type PP2Cδ but not phosphatase-dead mutant (D317A) was shown to abrogate hyperactivated phenotype of PP2Cδ^-/-^ DCs and the deregulated Th1/Th17 and Treg differentiation (**Figures S6A, B**). Together, our data demonstrated that PP2Cδ phosphatase promoted the ubiquitination and degradation of NSD2 *via* the CUL4 E3 ligase, thereby limiting the Rictor/mTORC2 pathway to control DC activation and functionality.

### PP2Cδ Mediates Metabolic-Epigenetic Regulation of DCs Through the mTORC2/ACLY Pathway

**Figure 6:**
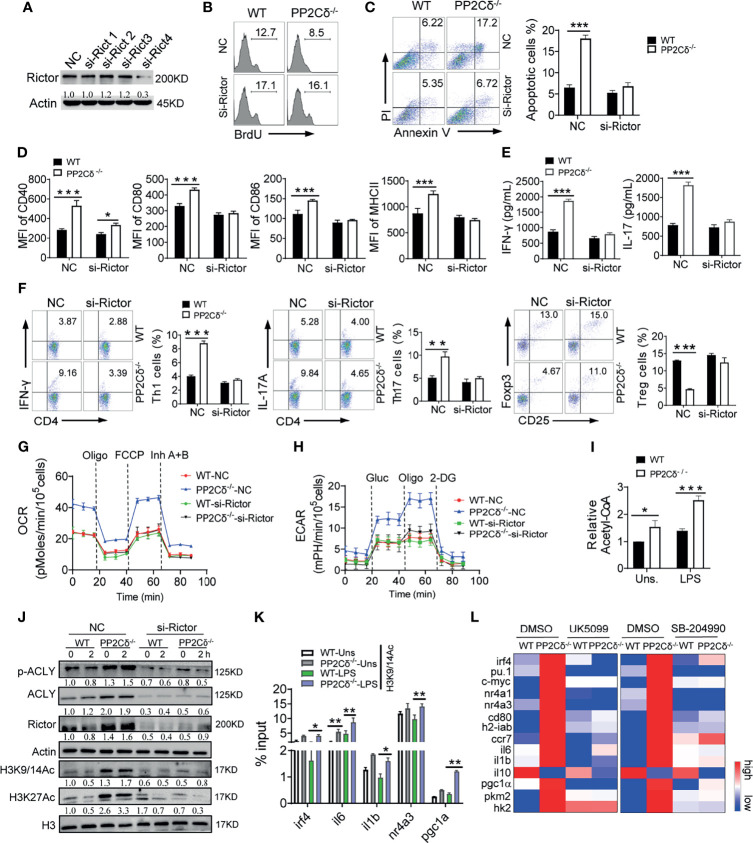
PP2Cδ coordinates DCs homeostasis through Rictor/mTORC2/ACLY pathway. **(A)** Immunoblotting of Rictor in DCs transfected with pre-designed siRNA (#1-4) for 48 h. **(B-H)** WT or PP2Cδ^-/-^ DCs were transfected with Rictor siRNA or non-specific control nucleotides (NC), followed by LPS stimulation. DCs were then subjected to the analysis of proliferation **(B)**, apoptosis **(C)**, activation markers expression **(D)**; T cell-secreted cytokines **(E)**; induction of OT-II T cells differentiation **(F)**; seahorse examination of OCR **(G)** and ECAR **(H)**; **(I)** Measurement of intracellular Acetyl-CoA in WT and PP2C^-/-^ DCs with or without LPS stimulation; **(J)** Immunoblotting of the indicated molecules in WT or PP2Cδ^-/-^ DCs transfected with Rictor-siRNA or NC for 48 h, followed by LPS stimulation for 0 or 2 h; The data below the lanes represent the bands densities relative to that of the loading control Actin. **(K)** ChIP assay of the indicated genes enrichment at the H3K9/14Ac in WT or PP2Cδ^-/-^ DCs with or without LPS stimulation. **(L)** Heatmapping of the indicated molecules in WT or PP2Cδ^-/-^ DCs with treatment of DMSO, UK5099 or SB-204990. Shown are representative images and the data from two or three independent experiments are expressed as means ± SD, with two or three technical replicates. *P < 0.05, **P < 0.01, ***P < 0.001 by student’s *t* test.

The activation of DCs is a coordinated process that integrates immunological signaling and metabolic program. Since mTOR pathway plays a central role in regulating cellular metabolism and differentiation, we thus further explored how the PP2Cδ/mTORC2 axis orchestrated the metabolic and gene programs that specified DC identity. We initially tested whether the Rictor/mTORC2 pathway was integrated into the DC regulatory program. Indeed, deletion of Rictor was shown to rectify the hyperactivated phenotype of PP2Cδ^-/-^ DCs, as revealed by the substantial alteration in cellular proliferation, apoptotic rate, activation markers levels, and Th1/Th17-stimulatory activity (**Figures 6A–F** and **Figure S7A**). Moreover, knockdown of Rictor decreased mitochondrial OXPHOS and glycolytic metabolism of PP2Cδ^-/-^ DCs to the level comparable to that in WT cells (**Figures 6G, H**). The data thus indicated that the Rictor/mTORC2 pathway served as the signaling nexus to link metabolic and effector programs of DCs, which prompted us to further explore the mechanism involved, particularly the metabolic-epigenetic pathway.

Recent studies have demonstrated that glycolytic byproducts such as pyruvate may through mitochondrial pyruvate carrier (MPC) enter mitochondria and be further catalyzed by ATP citrate lyase (ACLY) to generate acetyl-CoA (Ac-CoA). The generated Ac-CoA would be then transported to the nucleus for histone acetylation and genes transcription regulation (50, 51). This metabolic-epigenetic flux was thought to be driven by the mTORC2 and essential for genes expression that specified distinct subsets of immune cells (18, 22). Of interest, we observed a higher level of acetyl-CoA and increased phosphorylation of ACLY in PP2Cδ lacking DCs relative to WT cells, which, however, was reduced upon Rictor knockdown (**Figures 6I, J**). Along with this, the acetylation at H3K9/14 and H3K27, the transcription-permissive histone modifications (52), was augmented in PP2Cδ^-/-^ DCs, and Rictor deletion abolished this increment (**Figure 6J**). Consistent with this, boosted binding of H3Ac to the targeted genes was detected in PP2Cδ^-/-^ DCs relative to WT cells (**Figure 6K**). Thus, our data indicated that the Rictor/mTORC2 pathway activated ACLY and facilitated histone acetylation subsequently, which would contribute to PP2Cδ-mediated gene program in DCs. To further confirm this mechanistic linkage, we then applied UK5099 and SB204990 to block MPC or ACLY respectively. The results showed that either blocking mitochondrial pyruvate entry or inhibiting catalytic activity of ACLY corrected, though to different extent, the expression of genes characterizing hyperactivated DCs upon PP2Cδ deletion (**Figure 6L**). The results showed that a network of signature genes including the activation and metabolism-related genes (*il-6, il-1β, cd80, h2-iab*, and *ccr7*), mitochondrial and glycolytic metabolism-related genes (*pgc-1α, hk-2, and pkm2*), and DC-specifying transcription factors (*irf4, pu.1, c-myc, nr4a1*, and *nr4a3*) (53–55)were substantially altered upon these treatments. We thus proposed that ACLY-driven metabolic-epigenetic flux, downstream of the PP2Cδ/mTORC2 pathway, was critically involved in the gene program for DC identity. In addition, although previous study showed that lipid-derived acetyl-CoA could also fuel histone acetylation for gene resetting (56), our data indicated that PP2Cδ ablation did not affect the expression of genes essential for fatty acid metabolism (**Figure S7B**), implying that lipid metabolism might not the essential source of acetyl-CoA for epigenetic modification in our setting. Taken together, we showed that PP2Cδ acted through the Rictor/mTORC2/ACLY pathway to mediate the metabolic-epigenetic program, which was essential for activation and function of DCs (**Figure S7C**).

## Discussion

DCs have been established as a key player in the induction and maintenance of immune reaction or self-tolerance, and immunogenic or tolerogenic DCs are largely determined by their developing stage and maturing status (1, 57). Identifying key factors controlling DCs activity is therefore important for understanding the immune regulatory mechanism. In this study, we identify PP2Cδ as a key regulator of DCs activation and function through controlling mTORC2 pathway. PP2Cδ ablation remarkably strengthened the maturation, activation, and antigen-presenting activity of DCs, resulting in Th1/Th17-biased response and exaggerated EAE pathology. Mechanistically, PP2Cδ specifically associated with and de-phosphorylated the methyltransferase NSD2, facilitating its ubiquitination and proteolysis *via* the E3 ligase CUL4^DCAF2^, which in turn caused down-regulation of Rictor and hence mTORC2 signaling. By contrast, loss of PP2Cδ led to sustained activation of the Rictor/mTORC2 signaling and increased mitochondrial respiration and glycolytic metabolism, which not only yielded sufficient bioenergetics for hyperactivated DCs but also promoted ACLY-mediated epigenetic reprogramming of DCs. We thus establish PP2Cδ as an unappreciated checkpoint for DCs development and activation, and unravel an mTORC2-mediated metabolic-epigenetic program that is critical for DC biology.

Though initially conceived as an oncogene and a regulator of DNA damage response, PP2Cδ has been increasingly recognized as a key modulator for immune cell development and activity. Evidences have demonstrated that PP2Cδ plays a pivotal role in regulating the differentiation and function of a variety of immune cells including T cells, B cells, neutrophils, and macrophages (29–34). However, till now little is known about its role in DCs development and function. Our present study indicates that PP2Cδ constitutes a self-controlled mechanism to control DCs activation, maturation, and metabolism, and is therefore essential for preventing their excessive activation and induction of pathogenic T cell response. Of interest, PP2Cδ appears to be indispensable for steady-state development of DCs, as PP2Cδ deletion causes the decreased amounts of DCs developed from HSCs. This defect is likely associated with increased apoptotic rate and decreased proliferative ability of DCs upon PP2Cδ loss. In parallel, the genes key for cellular apoptosis and mitosis such as p53, Bax, Bcl2, and CDKs were aberrantly expressed in PP2Cδ^-/-^ DCs compared with their WT counterparts (**Figure S2C**). Additionally, PP2Cδ lacking DCs displayed increased mitochondrial ROS, which may also contribute to increased apoptosis of DCs (12). Intriguingly, we note that PP2Cδ^-/-^ DCs, though in higher level of cellular metabolism and ATP generation, exhibited blunted proliferative capability compared with WT cells. The plausible explanation for this might be that enhanced cellular metabolism and biosynthesis in PP2Cδ^-/-^ DCs might be used to support cellular activation rather than cellular expansion. DCs, upon activation, would rapidly undergo a functional shift with abundantly producing cytokines and surface markers, efficiently processing of antigens and migration to T cell zone. All of these activities require a large quantity of bioenergetics and biosynthesis, the processes essentially dependent on mTOR signaling (5–8).

On the other hand, functional reprogramming of DCs is generally accompanied epigenetic remolding, which is critical for shaping lineage or status-specific gene programs. As revealed in our study, the increased mTORC2 pathway upon PP2Cδ loss promoted the activation of ACLY, which in turn catalyzed acetyl-CoA generation for fueling the gene program compatible with hyperacted DCs. In this sense, the phosphatase PP2Cδ, through regulating mTORC2 pathway, serves to coordinate cellular metabolic and epigenetic program to control DCs fitness and homeostasis. Indeed, the pro-proliferation and pro-survival effects of PP2Cδ have been evidenced in HSC, neural stem/progenitor cells (NPCs), and B cells (32, 36, 58), indicating that the phosphatase mediates a general effect on cell fitness and sustenance.

As previous studies reported that mTORC1 was critically involved in PP2Cδ action during HSC and hepatocytes development (35, 36), we initially exploited rapamycin, the selective inhibitor of mTORC1, to examine how PP2Cδ modulates DC activity. Unexpectedly, the results showed that rapamycin treatment just moderately reduced the pro-inflammatory cytokines production by PP2Cδ-/- DCs following LPS stimulation, but marginally affected the levels of activation markers (data not shown). The results imply that mTORC1 might not be the principal substrate of PP2Cδ, or PP2Cδ mediates the DC regulatory role not exclusively through mTORC1 pathway. Notably, administration of mTORC1/2 inhibitor Torin1, or specific interference of Rictor in our system caused an almost complete abrogation of the effect imposed by PP2Cδ ablation, implicating the Rictor/mTORC2 signaling in PP2Cδ action. Importantly, our data unveil an unappreciated mechanism wherein PP2Cδ acts through the methyltransferase NSD2 to control Rictor and hence mTORC2 activity. In this scenario, PP2Cδ-mediated de-phosphorylation of NSD2 appears to be indispensable for its association with CRL4^DCAF2^, as phosphatase-dead mutant (D317A) of PP2Cδ precluded its binding to CRL4^DCAF2^ and the subsequent proteolysis. The findings thus link the post-translational regulation (phosphorylation, ubiquitination) with the epigenetic mechanism (methylation), and provide a self-controlled mechanism for DCs activity. Indeed, as a member of the protein Ser/Thr phosphatase family, PP2Cδ and its homologs have been demonstrated to couple the phosphorylation and epigenetic modifications to fine-tune the key signaling pathways. For instance, PP2Cδ was able to co-opt the acetyltransferase p300 for p53 acetylation and exert the regulatory effect on cell damage response (45). Also, the protein phosphatase PP2A dephosphorylated the demethylase Rph1 and inhibited its binding to histone for methylation, thereby regulating target genes expression. Interestingly, PP2A itself is also subjected to methylation depending on methionine availability, linking nutrient status with gene transcriptional machinery to control cell differentiation (59). In the present study, we for the first time identify the methyltransferase NSD2 as a substrate of PP2Cδ to mediate its regulation of mTORC2 pathway and hence DCs activity. NSD2 is a member of the nuclear receptor-binding SET domain protein (NSD) family capable of catalyzing the H3K36 methylation and remolding the chromatin configures to activate the genes key for cell proliferation, differentiation, and survival (60). Although presumed as a regulator of hematopoietic development and malignancy, NSD2 is increasingly recognized as a key regulator for immune cells such as T cells and B cells (61, 62). Our current study extends the regulatory spectrum of NSD2 by revealing its key role in DCs biology, and more critically, identifying it as a nexus factor that links PP2Cδ with the Rictor/mTORC2 pathway to exert immunoregulatory effect. Indeed, the enrichment of H3K36 demethylation was recently found to causatively relate with inflammatory cytokines release by DCs (63), conferring an additional support for our discovery. Additionally, NSD2 was reported to be phosphorylated and stabilized by Akt kinase, and epigenetically regulate Rictor to promote cancer metastasis (47), indicating that NSD2 exerts context-dependent effects. Thus, further studies might be merited to dissect how distinct signals like immune stimuli or oncogenic factors impinge the NSD2-driven mTOR signaling (64).

The development and activation program of DCs are shaped by metabolic rewiring and gene editing in response to immunological signals (65). It is currently recognized that, upon stimulation with pathogenic agents or danger signals, BMDCs induce a metabolic switch from oxidative phosphorylation (OXPHOS) to glycolysis, followed by the activation of anabolic pathways like fatty acid (FA) synthesis (12, 44, 66). The metabolic rewiring is critical for optimized DCs activation, because it not only offers bioenergy and biosynthetic products for cellular growth and function, but also generates metabolic intermediates for epigenetic modification. In the present study, we unravel that PP2Cδ, in addition to control DC activity, also functions as a cellular metabolic checkpoint by restraining anaerobic glycolysis and mitochondrial respiration. We note that mitochondria of PP2Cδ deficient DCs localized adjacent to endoplasmic reticulum (ER) and likely formed the so-called mitochondria-ER membrane (MAM). The observation is reminiscent of previous report that MAM was initiated upon mTORC2 activation underpinning enhanced mitochondrial physiology (43). In line with this, PP2Cδ^-/-^ DCs exhibited higher respiration rate and ATP generation, which is compatible with their highly activated phenotypes. More importantly, the augmented mTORC2 pathway, along with the increased metabolic flux, enables the activation of ACLY to catabolize Acetyl-CoA generation from glucose-derived citrate (22, 51, 52, 67). This is an essential step for metabolic-epigenetic machinery for PP2Cδ-/- DCs, as specific inhibition of MPC1 or ACLY largely abolished the effect of PP2Cδ loss on DC-associated genes program. Although lipid-derived acetyl-CoA also constitutes global acetyl-CoA pool (56), our data show that PP2Cδ did no affected the expression of lipid-related genes. The findings may exclude the potential contribution of alternative source of acetyl-CoA in PP2Cδ pathway. Thus, it may be tentatively concluded that PP2Cδ mediates the epigenetic regulation of DCs through controlling both the rate-limiting enzyme ACLY and the acetyl donation from metabolic flux.

In conclusion, our current study establishes PP2Cδ as a central factor for DC activity through regulating the Rictor/mTORC2 pathway *via* NSD2. We also unveil the importance of the mTORC2/ACLY pathway in metabolic-epigenetic regulation of DC homeostasis.

## Data Availability Statement

The raw data supporting the conclusions of this article will be made available by the authors, without undue reservation.

## Ethics Statement

The animal study was reviewed and approved by Animal Care and Use Committee of Nanjing University of Chinese Medicine.

## Author Contributions

NL and SJ designed and performed most of the experiments, analyzed the data, and wrote the manuscript. ZL, XW, YK, LS and YD carried out the experiments. BW and TM provided experimental material and intellectual input. LYS conceived the study, supervised the study and revised the manuscript. All authors contributed to the article and approved the submitted version.

## Funding

This work was supported by National Natural Scientific Funds (81770014, 81991523, and 82000014), the National Key Research and Development Program Project (2018YFC1705900), and the priority academic program development of Jiangsu higher education institutions. 

## Conflict of Interest

The authors declare that the research was conducted in the absence of any commercial or financial relationships that could be construed as a potential conflict of interest.

## Publisher’s Note

All claims expressed in this article are solely those of the authors and do not necessarily represent those of their affiliated organizations, or those of the publisher, the editors and the reviewers. Any product that may be evaluated in this article, or claim that may be made by its manufacturer, is not guaranteed or endorsed by the publisher.
