# Supplementary material for: PP2Cδ Controls the Differentiation and Function of Dendritic Cells Through Regulating the NSD2/mTORC2/ACLY Pathway
Source: Front Immunol. 2022 Jan 7;12:751409. doi: 10.3389/fimmu.2021.751409 (PMC8777276; doi:10.3389/fimmu.2021.751409)
Supplement: Supplementary file 1 [file DataSheet_1.pdf]

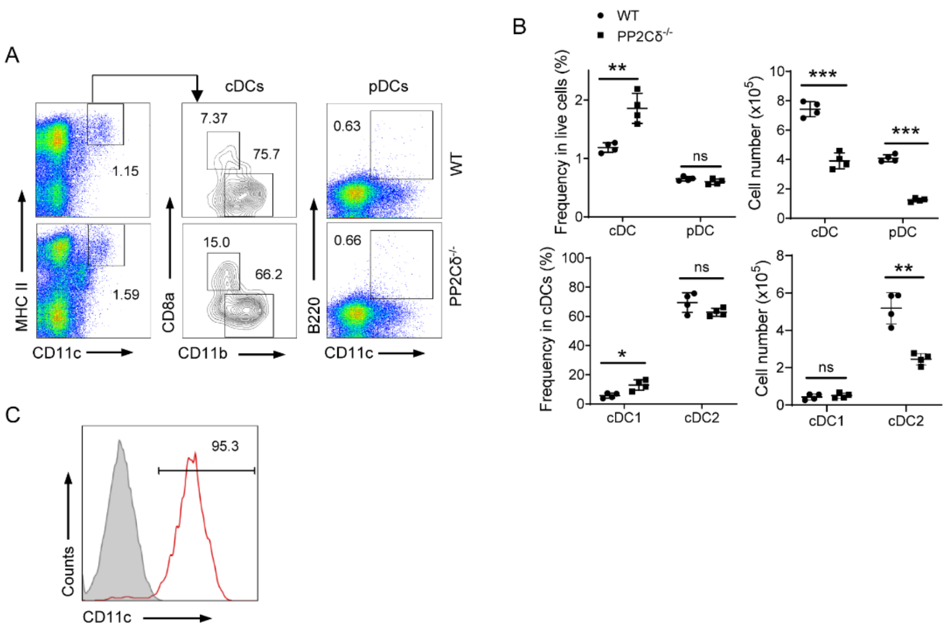

Fig. S1 The effect of PP2Cδ on DC development. **(A-B)** Flow cytometry analysis of splenic conventional DC (cDC) (A); pDC populations, cDC1 ($CD8\alpha^{+}CD11b^{-}$) and cDC2 ($CD8\alpha^{-}CD11b^{+}$) (B) in WT and PP2Cδ$^{-/-}$ mice. **(C)** Flow cytometry analysis of purity of CD11C^{+} BMDCs after enrichment. Shown are representative images and the data from two or three independent experiments are expressed as means ± SD. *P < 0.05, **P < 0.01, ***P < 0.001 by student's *t* test. ns, not significant.

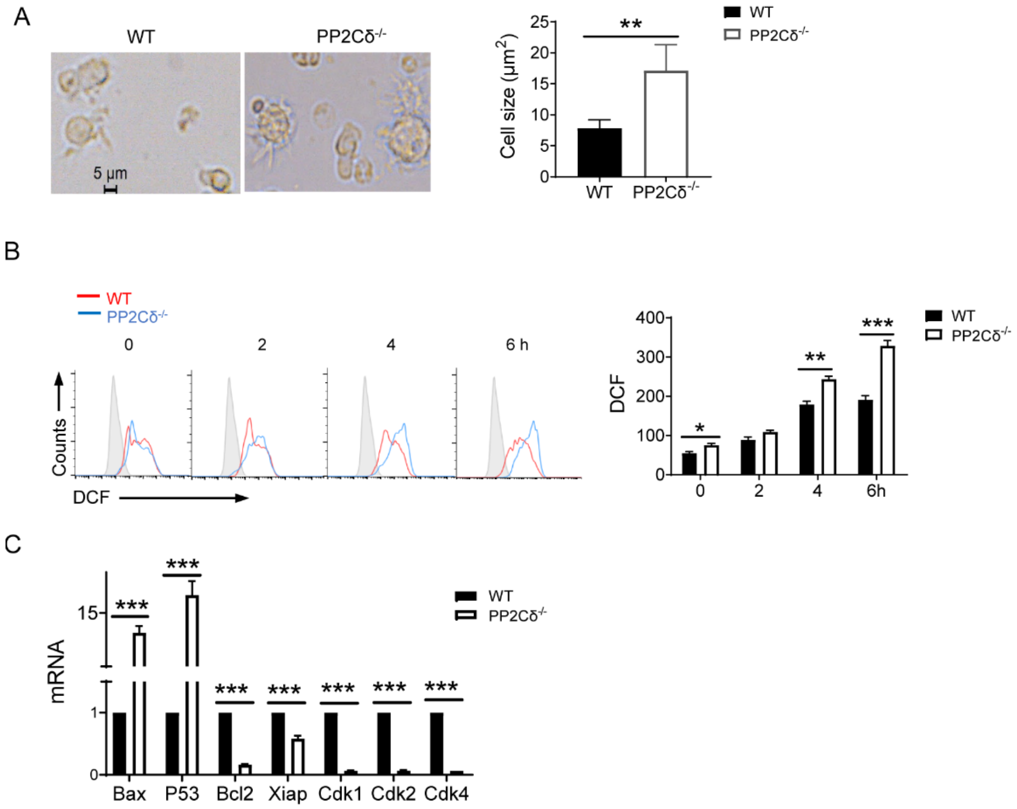

Fig. S2 PP2Cδ$^{-/-}$ DCs display enlarged cell size and increased ROS production. (A) Representative images showing morphology of BMDCs at day 6 culture; **(B)** Cellular ROS level in DCs with or without LPS (100 ng/mL) stimulation for the indicated time periods. **(C)** qPCR assay of apoptosis- and proliferation-associated molecules as indicated in WT and PP2Cδ$^{-/-}$ DCs. Shown are representative images and the data from three independent experiments are expressed as means ± SD. *P < 0.05, **P < 0.01, ***P < 0.001 by student's *t* test.

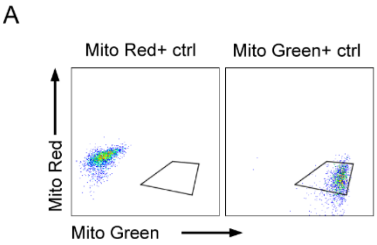

Fig. S3 Flow cytometry of mitochondria staining with MitoTracker Red and MitoTracker green. (A) DCs respectively stained with MitoTracker Red and MitoTracker green as controls.

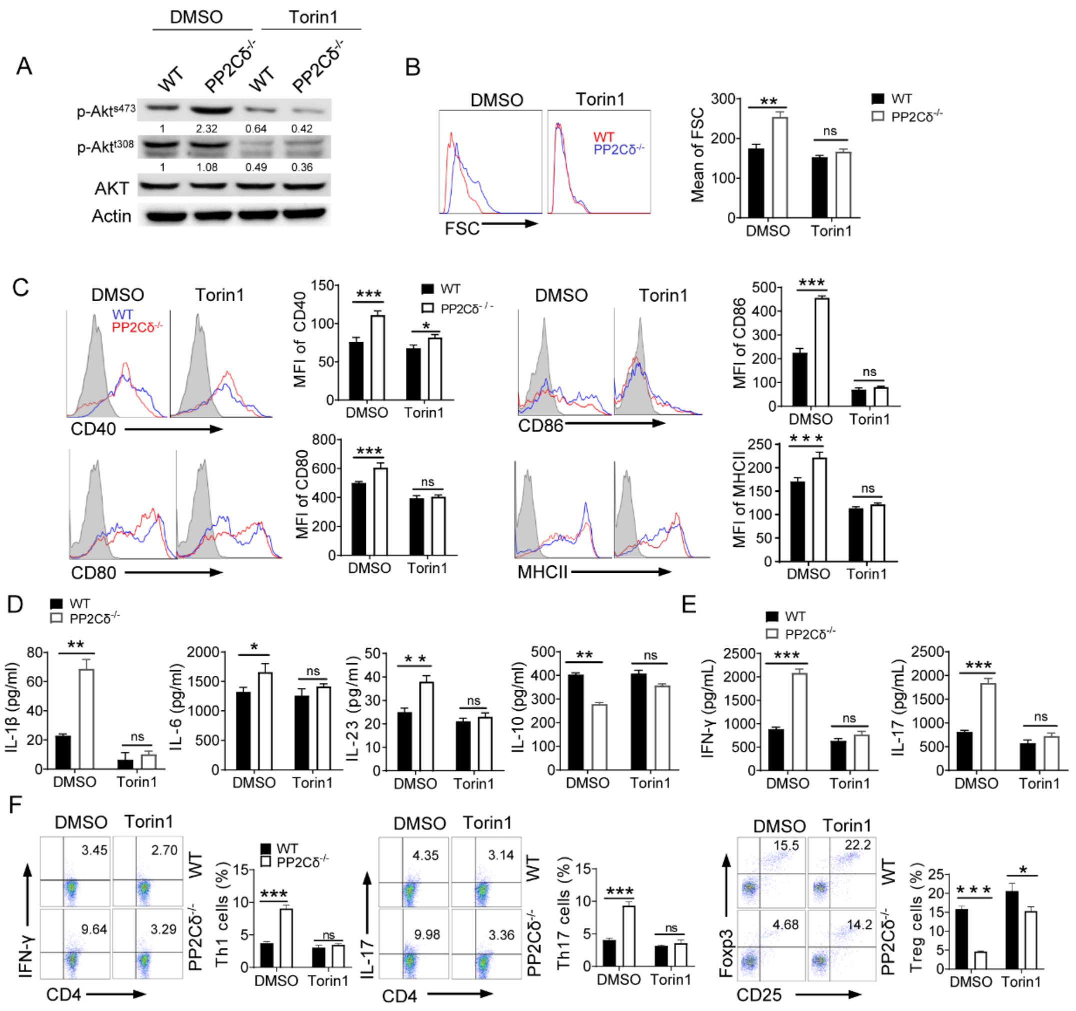

Fig. S4 Enhanced mTORC2 pathway underlies hyperactivated PP2Cδ$^{-/-}$ DCs. BMDCs from WT or PP2Cδ$^{-/-}$ mice were cultured in the presence of Torin1 (100 nM) or DMSO. **(A)** Immunoblotting analysis for the phosphorylated AKT after Torin1 treatment. The data represent the relative bands densities. **(B-F)** Cell size (B); Activation markers level (C) and cytokines production upon stimulation with LPS for 12 h (D); ELISA assay of levels of T cell-secreted cytokines (E); Induction of CD4+ T cells differentiation detected by intracellular staining (F). Shown are representative images and the data from three independent experiments are expressed as means ± SD. *P < 0.05, **P< 0.01, ***P < 0.001 by student's *t* test. ns, not significant.

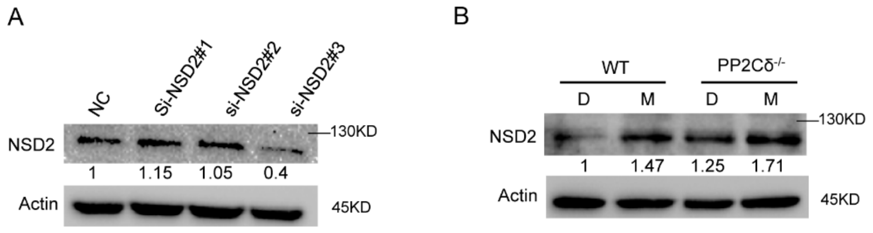

Fig. S5 Knockdown efficiency of NSD2 siRNA and the effect of proteasome inhibition on its expression. (A) Immunoblotting of NSD2 in DCs transfected with pre-designed siRNA (#1-3) for 48 h. **(B)** Immunoblotting of NSD2 in WT or PP2Cδ$^{-/-}$ DCs treated with MG132 (10 mM) or DMSO for 6 h. The data represent the bands densities relative to that of the loading control Actin. Shown are representative images from two experiments.

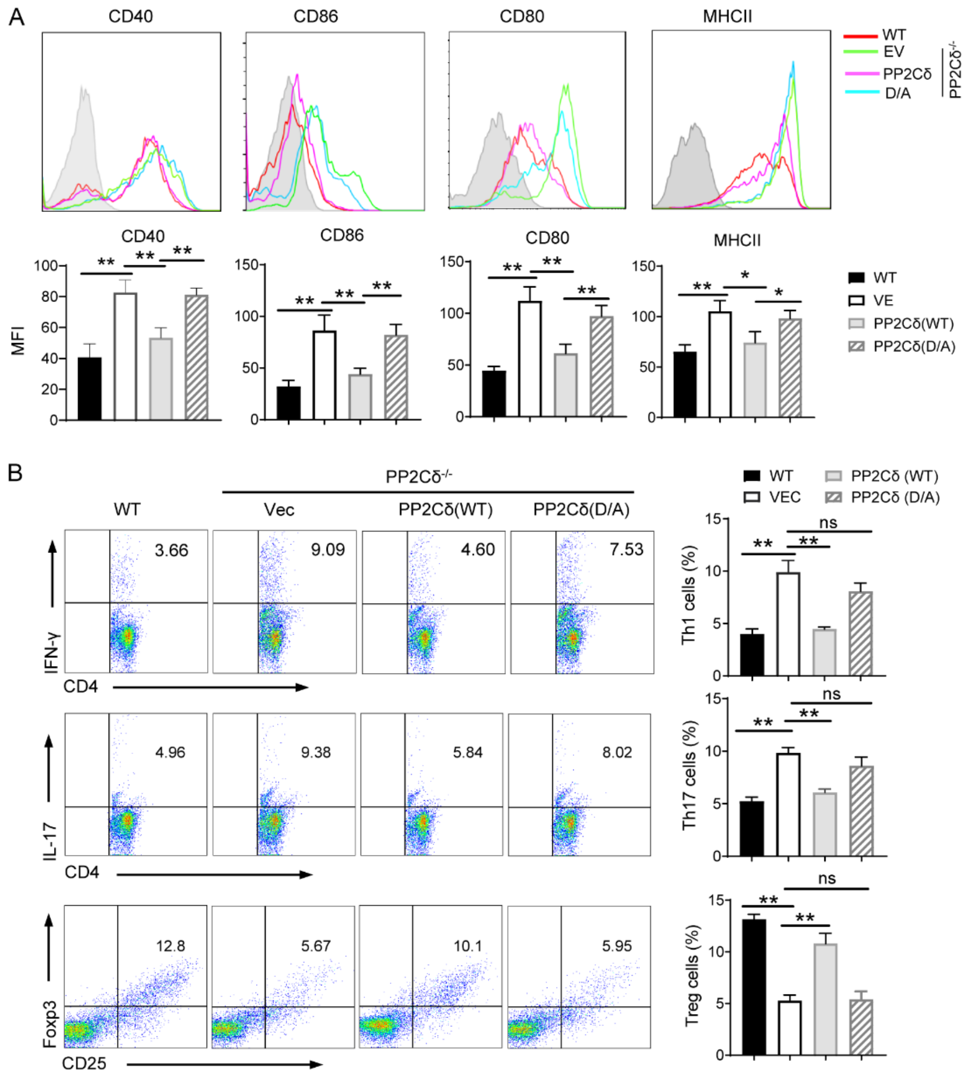

Fig. S6. Phosphatase activity of PP2Cδ is required for its DCs regulation. (A) Flow cytometry of activation markers levels in LPS-stimulated WT or PP2Cδ$^{-/-}$ DCs that were pre-transfected with empty plasmids, the intact or phosphatase-inactivated (D317A) PP2Cδ-expressing plasmids. **(B)** WT or PP2Cδ$^{-/-}$ DCs were stimulated with LPS (100 ng/mL) and pulsed with OVA peptide overnight, and then co-cultured with OT-II T cells. The frequencies of Th1, Th17 and Treg cells were detected by intracellular staining and flow cytometry analysis. Shown are representative images and the data from three independent experiments are expressed as means ± SD. *P < 0.05, **P < 0.01, ***P < 0.001, by student's *t* test. ns, not significant.

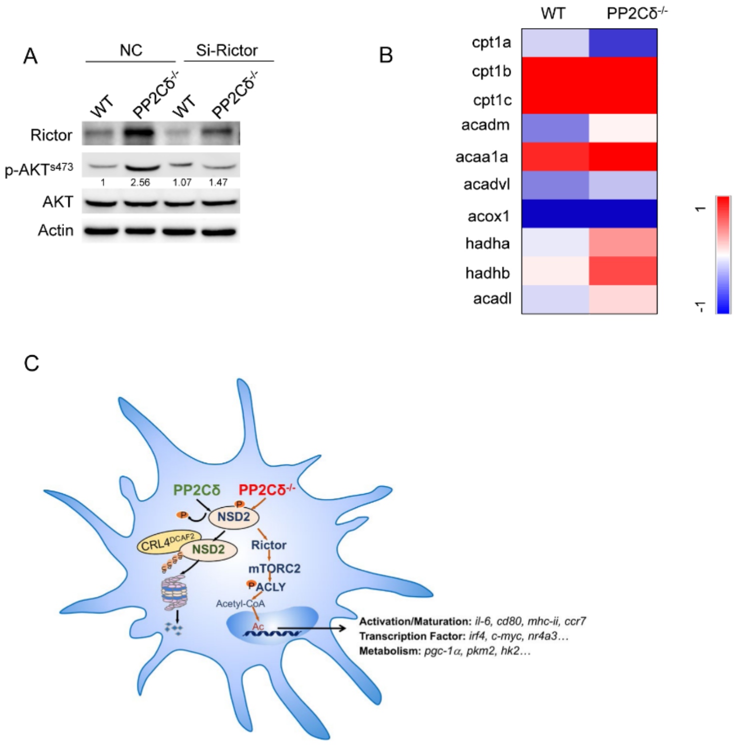

Fig. S7. Effect of PP2Cδ on the expression of genes involved in lipid metabolism.

(A) Immunoblotting analysis for the phosphorylated AKT after Rictor knockdown. The data represent the bands densities relative to that of the loading control Actin. **(B)** Heatmap showing the expression of fatty acid oxidation-associated genes in WT and PP2Cδ$^{-/-}$ DCs. **(C)** The proposed working model of PP2Cδ in DC regulation. Shown are representative data from two independent experiments.

Table S1: the primers of RT-PCR

Gene	Forward primer (5′–3′)	Reverse primer (5′–3′)
Il1β	TGCCACCTTTTGACAGTGATG	TGATGTGCTGCTGCGAGATT
Il6	GTCCTTCCTACCCCAATTTCCA	TAACGCACTAGGTTTGCCGA
Il12p35	AGTTTGGCCAGGGTCATTCC	TCTCTGGCCGTCTTCACCAT
Tnfα	CCTCACACTCACAAACCACCA	ACAAGGTACAACCCATCGGC
Il10	TTCATTGCATACGGGACAGAAC	TTTCCAGTGGAGGATGTGCT
Ndufa4	CTGGAGCAGCACTGTATGTGA	TTGGGACCCAGTTTGTTCCAT
Ndufa8	GAGTTTATGCTGTGCCGCTG	TACTCTGTGAAAGGCTCCGC
Ndufa11	TCCGCTTACAGCGTCTCAC	AGGCCAAACATCGCTCCAAT
Ndufb9	ACCGGTACTTTGCTTGCTTG	ATCTCTCGAAGGAAGTGCCC
Ndufv1	TGCTTGTGGCTCCGACTATG	ACAGTTGTGGGGCATCCAAA
Sdhb	CAGAGTCGGCCTGCAGTTT	ATCCAACACCATAGGTCCGC
Sdhd	CTGGTTCCAAGGCTGCATCT	AGCCAGAGAGTAGTCCACCA
Cyc1	ATCGTTCGAGCTAGGCATGG	GCCGGGAAAGTAAGGGTTGA
Uqcr11	GGAACTGGCCAGAAACTGGA	TGCCGTTGATGTAAGGCACC
Uqcrc1	ATGCTGCGTGACATTTGCTC	TAGAAGCGCAGCCAGAACAT
Cox6a1	CAACGTGTTCCTCAAGTCGC	CTTCATAGCCGGTCGGAAGT
Cox6b1	AGAACTACAAAACTGCCCCCT	TTCTCACAGCGGTGGAAGTC
Cox5a	TGTCTGTTCCATTCGCTGCT	AACCGTCTACATGCTCGCAA
Cox5b	GCTTCAAGGTTACTTCGCGG	ATGGGTCCAGTCCCTTCTGT
Cox7c	GAGTATCCGGAGGTTCACGAC	ACCGCCACTTGTTTTCCACT
Cox8a	CAGGTCCACTCGAAGCCG	CAGGCAGAAGACAACACACG
Cox15	GCGTCCGGCAACGGT	TGATGGTGCTGTACTGTCCT
Atp5d	TACGCTGACTGGAGCCTTTG	GTCCAGCATGTCCAGTGTCA
Atp5g1	GCCAAGTTCATTGGTGCTGG	GGAGAAGAGCTGCTGCTTGA
Atp5g2	ATGTACGCCTGCTCCAAGTT	CTGTGGTCGCTTCAACTCCA
Atp5g3	CAGCTGATCCGAAGGGAGTTT	TGAAGGGTTTCAGCACCAGAA
Atp5h	TGGAATGAGACCTTCCACGC	GCACAGGAATCTTCAGGGCA
Atp5j2	GAGCTGCCGAGCTGGATAAT	GGACCATGCTAATCCCCGAG
Atp5k	GGTTCAGGTCTCTCCACTCA	CTCCGCTGCTATTCTCCTCTC
Atp5e	TCAGCTACATCCGGTTTTCCC	TTTTATGCTGCTGCCCGAAG
Atp6v1	ACATCGCAGAGATGGTTCGG	CTTTGGCTGCATCGTAGGGA
Atp6v0c	CATCGTCGGAGATGCTGGTG	GGAGAGGATTAGGGCCACGA
Hif1a	GGGGAGGACGATGAACATCAA	GGGTGGTTTCTTGTACCCACA
Gapdh	AGGTCGGTGTGAACGGATTTG	GGGGTCGTTGATGGCAACA
Ldh5a	CATTGTCAAGTACAGTCCACACT	TTCCAATTACTCGGTTTTTGGGA
Pkm2	GTGGCTCGGCTGAATTTCTCT	CACCGCAACAGGACGGTAG
Hk2	GATTTCACCAAGCGTGGACT	CCACACCCACTGTCACTTTG
Tpi1	TGCATCGGGGAGAAGCTAGA	TGGCCCACACAGGTTCATAG
Eno1	GAGGTCGATCTGTACACCGC	ACTTTCTGAGTGCACAAGGCT
Aldoa	CACCCAGCAACAGACAGAGT	AATGCAGGGATTCACACGGT
Gpi	TTGTCGCCCTGTCTACGAAC	CTCACAGCCGTAGCAGTTGA
Glut1	GATCCCAGCAGCAAGAAGGT	AGAGACCAAAGCGTGGTGAG

Gene	Forward primer (5′–3′)	Reverse primer (5′–3′)
Irf4	CCGACAGTGGTTGATCGACC	CCTCACGATTGTAGTCCTGCTT
Pu.1	ATGTTACAGGCGTGCAAAATGG	TGATCGCTATGGCTTTCTCCA
c-myc	GAGCTGTTTGAAGGCTGGATTT	TCCTGTTGGTGAAGTTCACGTT
Nr4a1	TTGAGTTCGGCAAGCCTACC	GTGTACCCGTCCATGAAGGTG
Nr4a3	TATGGCTCGGAATACACCACA	GCCCTCCATGAAGGTACTGAA
Cd80	TGCTGCTGATTCGTCTTTCAC	GAGGAGAGTTGTAACGGCAAG
H2-iab	ACAGCTTATTAGGAATGGGGACT	CACGGTGATGGGACTCTTCA
Ccr7	TGTACGAGTCGGTGTGCTTC	GGTAGGTATCCGTCATGGTCTTG
Pgc1a	GCAGCCAAGACTCTGTATGG	TTCCGATTGGTCGCTACACC
Bax	TGCTAGCAAACTGGTGCTCA	CCAGCCACCCTGGTCTTG
P53	CCCCTGTCATCTTTTGTCCCT	AGCTGGCAGAATAGCTTATTGAG
Bcl2	TCTTTGAGTTCGGTGGGGTC	AGTTCCACAAAGGCATCCCAG
Xiap	CTGGCCGGACTATGCTCATT	CACGATCACAGGGTTCCCAA
Cdk1	ACGGCGACTCAGAGATTGAC	GGCTTCCACTTGGGAAAGGT
Cdk2	GTGGTACCGAGCACCTGAAA	CGGGTCACCATTTCAGCAAA
Cdk4	GACGGTGTACAAAGCCCGA	CAAGGCCACCTCACGAACT
mtDNA region 1	TGAACGGCTAAACGAGGGTC	AGCTCCATAGGGTCTTCTCGT
mtDNA region 2	CAGTCCCCTCCCTAGGACTT	ACCCTGGTCGGTTTGATGTT
mtDNA region 3	TAATCGCACATGGCCTCACA	GAAGTCCTCGGGCCATGATT
gDNA B2m	AGCAAAGAGGCCTAATTGAAGTC	GAAGTAGCCACAGGGTTGGG
Rictor-siRNA	GCGAGCUGAUGUAGAAUUGTT	CAAUUCUACAUCAGCUCGCTT
NSD2-siRNA	GCCAGUAUCAUGUACAGUUTT	AACUGUACAUGAUACUGGCTT
